# Maternal alcohol consumption during pregnancy and child development: Role of *ADH1B* and *ALDH2* gene polymorphisms—The Yamanashi Adjunct Study of the Japan Environment and Children's Study

**DOI:** 10.1111/acer.15487

**Published:** 2024-11-13

**Authors:** Kunio Miyake, Sanae Otawa, Megumi Kushima, Hideki Yui, Ryoji Shinohara, Sayaka Horiuchi, Yuka Akiyama, Tadao Ooka, Reiji Kojima, Hiroshi Yokomichi, Zentaro Yamagata, The Yamanashi Adjunct Study of the Japan Environment and Children's Study Group, Anna Kobayashi, Takeshi Inukai, Kyoichiro Tsuchiya, Hirotaka Haro, Masanori Wako, Takahiko Mitsui, Kenji Kashiwagi, Daijyu Sakurai, Koichiro Ueki, Sumire Ono

**Affiliations:** ^1^ Department of Epidemiology and Environmental Medicine, Interdisciplinary Graduate School of Medicine and Engineering University of Yamanashi Chuo Japan; ^2^ Center for Birth Cohort Studies University of Yamanashi Chuo Japan; ^3^ Department of Health Sciences, Interdisciplinary Graduate School of Medicine and Engineering University of Yamanashi Chuo Japan

**Keywords:** *ADH1B*, alcohol, *ALDH2*, ASQ‐3, pregnant women

## Abstract

**Background:**

The role of polymorphisms in genes regulating alcohol metabolism, particularly those modulating the impact of prenatal alcohol exposure on the neurodevelopment of offspring, remains inconclusive. Herein, we aimed to determine the involvement of *ADH1B* and *ALDH2* gene polymorphisms in maternal alcohol consumption during pregnancy and the risk of developmental delay in offspring in a Japanese population.

**Method**s**:**

We analyzed 1727 mother–child pairs from the Yamanashi Adjunct Study of the Japan Environment and Children's Study. Maternal alcohol consumption during pregnancy was determined through a mid‐pregnancy questionnaire and categorized into three groups: never‐drinkers, those who quit drinking in early pregnancy, and current drinkers. Developmental delays in children were assessed in five domains using the Japanese version of the Ages and Stages Questionnaire, Third Edition (J‐ASQ‐3) at 3 years of age. We conducted a logistic regression analysis to explore the relationship between maternal drinking status during pregnancy and developmental delays in offspring with respect to maternal *ADH1B* (rs1229984) or *ALDH2* (rs671) gene polymorphisms.

**Results:**

Children born to mothers who continued alcohol consumption during pregnancy had a higher risk of delayed communication skills at 3 years of age compared with children born to mothers who did not drink alcohol (adjusted odds ratio [OR], 5.82; 95% confidence interval, 1.84–18.38). Analysis by *ALDH2* gene polymorphism revealed that alcohol consumption by mothers carrying the wild‐type *ALDH2* (*1/*1) increased the risk of delayed communication skills at 3 years of age, whereas alcohol consumption by mothers carrying a heterozygotic genotype of *ALDH2* (*1/*2) enhanced the risk of developmental delay in all five domains of the J‐ASQ‐3. The impact of *ADH1B* gene polymorphism could not be clearly elucidated.

**Conclusions:**

Our results suggest that alcohol consumption by pregnant females carrying the deficient variant ALDH2*2 genotype may increase the risk of developmental delay in their offspring.

## Graphical abstract

This study aimed to elucidate the impact of maternal alcohol consumption during pregnancy on developmental delays in offspring, considering the polymorphisms of *ADH1B* and *ALDH2*, using a Japanese birth cohort study. Maternal alcohol consumption during pregnancy was associated with an increased risk of communication delays at 3 years of age. Alcohol consumption during pregnancy in mothers with the ALDH2*1/*2 genotype was found to increase the risk of developmental delays in all domains of the J‐ASQ‐3.

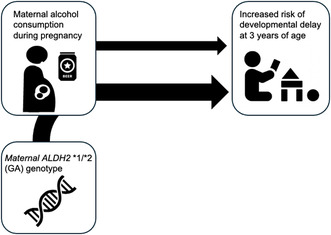

## INTRODUCTION

Prenatal exposure to alcohol is known to cause fetal alcohol spectrum disorder (FASD). FASD includes a wide range of physical, cognitive, and behavioral problems, with fetal alcohol syndrome (FAS) identified as the most severe manifestation, resulting in characteristic facial features, developmental delays, and central nervous system dysfunction (Denny et al., 2017; Jones & Smith, 1973). Even in the absence of the typical symptoms of FAS, prenatal exposure to alcohol may negatively impact the child's neurodevelopment, leading to attention deficits, learning delays, poor memory, and impaired social skills (Du Plooy et al., 2016; O'Callaghan et al., 2007; Panczakiewicz et al., 2016).

According to data acquired in 2012, 9.8% of women consume alcohol during pregnancy worldwide, with an estimated 25.2% in the European region (Popova et al., 2017). In Japan, the percentage of mothers who consume alcohol during pregnancy is declining, plummeting to 1.0% in 2019 (Health Japan 21, 2023). Currently, the safe level of alcohol intake during pregnancy remains unknown. Recent studies have revealed that even low‐to‐moderate alcohol intake during pregnancy may increase the risk of FASD (Chambers et al., 2019; Kesmodel et al., 2019). Thus, guidelines from countries worldwide emphasize the importance of avoiding alcohol consumption by females who are pregnant, planning to become pregnant, and breastfeeding.

Alcohol is primarily metabolized by alcohol dehydrogenase (ADH) and aldehyde dehydrogenase (ALDH). The most studied genetic variants in alcohol metabolism include *ADH1B* and *ALDH2* (Eng et al., 2007). Both rs1229984 (ADH1B*1 and *2) and rs2066702 (ADH1B*3) are single‐nucleotide polymorphisms (SNPs) of *ADH1B* (Polimanti & Gelernter, 2018). The ADH1B*1 allele produces a protein with arginine at positions 48 and 370, which exhibits the lowest enzymatic activity among the three isozymes with respect to ethanol metabolism. Conversely, the ADH1B*2 allele results in a protein with histidine at position 48 and arginine at position 370, contributing to heightened enzyme activity. This increased activity facilitates the rapid clearance of ethanol. Furthermore, the protein product of the ADH1B*3 allele, characterized by arginine at position 48 and cysteine at position 370, similarly demonstrates prompt ethanol clearance.

*ALDH2* (rs671) has a wild‐type ALDH2*1 allele and an inactive ALDH2*2 allele. The ALDH2*2 allele leads to a protein product in which lysine replaces glutamine at position 487. ALDH2*2 homozygotes have almost no activity. Enzymatic activity in ALDH2*2 heterozygotes is reportedly less than 20% when compared with that of ALDH2*1 homozygotes (Edenberg & McClintick, 2018; Kitagawa et al., 2000). The allele frequencies of these genes vary by ancestral populations; ADH1B*1 is most prevalent in non‐Hispanic Whites and Blacks or African Americans, while ADH1B*2 is frequently detected in Northeast Asians. ADH1B*3 is predominantly found in individuals of African descent, and ALDH2*2 is almost exclusively present in Northeast Asians (Brennan et al., 2004; Edenberg & McClintick, 2018; Wall et al., 2016).

The effects of maternal alcohol consumption during pregnancy on the fetus are thought to vary substantially depending on maternal polymorphisms in genes regulating alcohol metabolism. Several studies conducted in the United States and South Africa nearly 20 years ago primarily focused on the *ADH1B* rs1229984 polymorphisms. In most of these studies, homozygosity for ADH1B*1 was found to be associated with an increased risk of FAS (Green & Stoler, 2007). However, recent studies in Poland and the UK have shown that genetic polymorphisms in *ADH1B* (rs1229984 and rs1789891) are not associated with the risk of FASD (Howe et al., 2019; Kukowka et al., 2023). These studies focused on the effects of increased ethanol exposure on the fetus owing to decreased ADH1B activity. On the contrary, few studies have involved Northeast Asians, who are known to have a high prevalence of the ALDH2*2 allele, and the effects of increased acetaldehyde owing to decreased ALDH2 activity remain largely unknown. Therefore, in the current study, we aimed to determine the effects of maternal alcohol consumption during pregnancy on delayed development in the offspring with respect to *ADH1B* (rs1229984) and *ALDH2* (rs671) polymorphisms using a Japanese birth cohort.

## MATERIALS AND METHODS

### Study setting and population

The Japan Environment and Children's Study (JECS) is a nationwide birth cohort study planned by the JECS Working Group to elucidate environmental factors that influence the health and development of children. Pregnant women were recruited from January 2011 to March 2014, resulting in over 100,000 participants in the JECS. The detailed protocols have been published elsewhere (Kawamoto et al., 2014; Michikawa et al., 2018). The dataset utilized in this study is the jecs‐ta‐20190930‐qsn, initially released in October 2019 and finalized in March 2022. The JECS comprises 15 regional centers spanning 19 prefectures throughout Japan, with the current study concentrating on the Koshin Regional Center, which includes Yamanashi and Nagano prefectures. Between 2019 and 2022, additional surveys were conducted among participants in Yamanashi Prefecture when the respective children turned 8 years of age, with blood samples collected from both mothers and children for DNA analysis. Of the 2036 additional survey participants, we excluded those with a maternal history of neurodevelopmental disorders, those with missing data on maternal drinking status during pregnancy, and those with missing information on maternal SNPs and the Japanese version of the Ages and Stages Questionnaire, Third Edition (J‐ASQ‐3), scores of the offspring at 3 years. Ultimately, our analysis focused on 1727 mother–child pairs in the current study. The JECS protocol was reviewed and approved by the Ministry of the Environment's Institutional Review Board for Epidemiological Studies and the Ethics Committees of all participating institutions (ethical approval number: 100910001). Additionally, this study was approved by the Institutional Review Board of Yamanashi University (No.: 2070, 2218). The research adhered to the principles of the Declaration of Helsinki, and explicit written consent was acquired from each participant involved in the study.

### Alcohol consumption status

Maternal drinking status was ascertained during the second/third trimester using a self‐administered dietary assessment tool, the food frequency questionnaire, which has been validated previously (Yokoyama et al., 2016). Each mother was instructed to select from one of the following options: “Never;” “Previously did, but quit;” “Previously did, but quit before recognizing the current pregnancy;” “Previously did, but quit after finding out about current pregnancy;” or “I'm still drinking.” In the current study, we re‐grouped these options into three categories: “Never,” “Quit drinking in early pregnancy,” and “Current drinkers.”

Mothers who reported current alcohol consumption were specifically asked about the frequency, types, and amount of alcohol consumed. The mother's drinking frequency was assessed using the following question: “Since you found out you were pregnant, how often have you drunk alcohol?” The options for the responses were as follows: “Hardly drank,” “1 to 3 times a month,” “1 to 2 times a week,” “3 to 4 times a week,” “5 to 6 times a week,” and “I drink every day.” Subsequently, we re‐stratified these options into three categories: “Hardly drank alcohol,” “1–3 times a month,” and “1–2 times a week or more.” The amount of alcohol consumed was assessed using the question, “Since you found out you were pregnant, how much alcohol did you drink?” The alcohol content of each beverage (sake, distilled spirits, beer, whiskey, and wine) was summed to calculate the total ethanol exposure (g/week). One standard drink was defined as containing 14 g of ethanol (Kurita et al., 2021). Mothers who were not currently drinking were categorized as “Never.”

The maternal drinking status after birth was determined using a questionnaire administered when the child was 1.5 years of age. Respondents selected from among the following options to determine the frequency of drinking: “seldom,” “1 to 3 times a month,” “1 to 2 times a week,” “3 to 4 times a week,” “5 to 6 times a week,” and “every day.” Herein, we re‐categorized the frequency of postdelivery drinking into four categories: “hardly drink,” “1–3 times a month,” “1–2 times a week,” and “3 times a week or more.”

### Genotyping

Genomic DNA was extracted from maternal whole blood using the FlexiGene DNA kit (Qiagen). The *ADH1B* (rs1229984) and *ALDH2* (rs671) genotypes were determined using the Biomark HD system (Standard BioTools) according to the manufacturer's protocol.

### Outcome definitions

Developmental delays in the offspring were evaluated using the J‐ASQ‐3, which was included in a questionnaire sent to the participants when the offspring turned 3 years of age. The J‐ASQ‐3 comprises a total of 30 questions, with six questions in each of the five domains: communication, gross motor, fine motor, problem‐solving, and personal and social skills. Parents/caregivers were required to select one of the following for each question: “Yes” if their child can perform the activity; “Sometimes” if their child can occasionally perform the activity; or “No yet” if their child cannot perform the activity. Each response of “Yes,” “Sometimes,” and “No yet” was assigned 10, 5, and 0 points, respectively.

The total score for each domain ranged between 0 and 60 points. If the score fell below the cutoff for each domain, the child was considered to have developmental delays in that domain. The cutoff scores for the J‐ASQ‐3 have been validated for Japanese children, and cutoff scores for developmental delay at 3 years of age are reported as follows: communication (29.95), gross motor skills (39.26), fine motor skills (27.91), problem‐solving (30.03), and personal–social (29.89) (Mezawa et al., 2019).

### Covariates

We selected confounding factors associated with drinking habits and those that affect child development based on the previous literature (Black et al., 2017; Little et al., 2002; Maher et al., 2022; Miyake et al., 2023). Information regarding maternal smoking during pregnancy, annual household income (million yen), maternal educational level, and maternal age at birth was collected from mothers in the first and second/third trimesters of pregnancy using a self‐administered questionnaire. Child sex, prepregnancy body mass index (BMI), and birth weight were extracted from medical record transcripts. Breastfeeding information was gathered using a self‐administered questionnaire when the child was 1 year of age. Maternal age at birth was categorized as <25 years, 25–29 years, 30–34 years, and >35 years. The prepregnancy BMI was categorized into the underweight (<18.5 kg/m^2^), normal (18.5–24.9 kg/m^2^), and overweight (≥25 kg/m^2^) groups.

### Statistical analyses

We examined whether maternal alcohol consumption during pregnancy was associated with developmental delays at the age of 3 years using multivariate logistic regression analysis. We utilized the group of mothers who did not drink alcohol during pregnancy as the reference and calculated the odds ratios (ORs) for developmental delay risk in other groups. In the multivariable models, adjustments were made for prepregnancy BMI, maternal age at birth, and breastfeeding until the child reached 1 year of age. Both crude ORs (cOR) and adjusted ORs (aOR), along with their respective 95% confidence intervals (CI), were calculated.

To elucidate the interaction between alcohol consumption and genetic polymorphisms, participants were stratified based on maternal drinking status during pregnancy and the combination of *ADH1B* or *ALDH2* genotype, and a multivariate logistic regression analysis was performed. The *ADH1B* genotype was classified into *2/*2 and *1/*1 + *1/*2. Given the absence of any mother who had the ALDH2*2/*2 genotype and drank alcohol during pregnancy, mothers with the ALDH2*2/*2 genotype were excluded from the analysis. Ultimately, a total of six categories were established, representing the combination of maternal drinking status during pregnancy (three groups) with either *ADH1B* genotype (two types) or *ALDH2* genotype (two types). Subsequently, logistic regression analysis was performed to explore the association between these exposures and developmental delay at the age of 3 years. The interaction between maternal drinking status during pregnancy and the *ADH1B* or *ALDH2* genotypes was assessed using the Wald test for the product term. We calculated the ORs for developmental delay in other groups when compared with a reference group of mothers who did not drink alcohol during pregnancy and had active gene variants (ADH1B*2/*2 or ALDH2*1/*1).

Missing values were excluded from the multivariate analysis. Statistical significance was set at a *P*‐value of 0.05 (two‐sided). All statistical analyses were conducted using the Statistical Package for the Social Sciences software version 27.0 (IBM Corp., Armonk, NY, USA).

## RESULTS

Table 1 summarizes the characteristics of the mothers and their children. The maternal drinking status during pregnancy was as follows: 958 mothers (55.5%) did not drink alcohol before becoming pregnant; 735 mothers (42.6%) stopped drinking after finding out that they were pregnant; and 34 mothers (2.0%) continued drinking during pregnancy. The number of 3‐year‐olds exhibiting scores below the cutoff value for each J‐ASQ‐3 domain were as follows: communication, *n* = 41 (2.4%); gross motor skills, *n* = 78 (4.5%); fine motor skills, *n* = 107 (6.2%) problem‐solving skills, *n* = 102 (5.9%); and personal–social skills, *n* = 42 (2.4%).

**TABLE 1:** Characteristics of the study population.

	All, 1727 (100)	Maternal drinking status during pregnancy		
		Never, 958 (55.5)	Quit drinking in early pregnancy, 735 (42.6)	Current drinking, 34 (2.0)
Maternal age at birth (years)				
<25	81 (4.7)	47 (2.7)	34 (2.0)	0 (0.0)
25–29	411 (23.8)	234 (13.5)	169 (9.8)	8 (0.5)
30–34	621 (36.0)	352 (20.4)	260 (15.1)	9 (0.5)
≥35	614 (35.6)	325 (18.8)	272 (15.7)	17 (1.0)
Maternal smoking during pregnancy				
No	1678 (98.0)	932 (54.4)	713 (41.6)	33 (1.9)
Yes	35 (2.0)	16 (0.9)	18 (1.1)	1 (0.1)
Missing data	14 (0.8)			
Prepregnancy BMI (kg/m^2^)				
<18.5	314 (18.2)	182 (10.5)	129 (7.5)	3 (0.2)
18.5–25	1260 (73.0)	690 (40.0)	540 (31.3)	30 (1.7)
>25	153 (8.9)	86 (5.0)	66 (3.8)	1 (0.1)
Annual household income (million yen)				
<4	611 (37.1)	347 (21.1)	248 (15.1)	16 (1.0)
4–6	525 (31.9)	292 (17.7)	224 (13.6)	9 (0.5)
≥6	510 (31.0)	273 (16.6)	229 (13.9)	8 (0.5)
Missing data	81 (4.7)			
Birth weight (g)	2991.3 ± 414.9	2985.1 ± 424.1	2999.5 ± 401.8	2988.4 ± 437.9
Sex of child				
Male	832 (48.2)	442 (25.6)	375 (21.7)	15 (0.9)
Female	895 (51.8)	516 (29.9)	360 (20.8)	19 (1.1)
Older siblings				
No	919 (53.2)	461 (26.7)	449 (26.0)	9 (0.5)
Yes	808 (46.8)	497 (28.8)	286 (16.6)	25 (1.4)
Breastfeeding at 1 year of age				
No	657 (38.6)	336 (19.8)	310 (18.2)	11 (0.6)
Yes	1044 (61.4)	604 (35.3)	417 (24.5)	23 (1.4)
Missing data	26 (1.5)			
J‐ASQ‐3 score at age 3 years of age				
Communication skill				
≥29.95	1686 (97.6)	936 (54.2)	720 (41.7)	30 (1.7)
<29.95	41 (2.4)	22 (1.3)	15 (0.9)	4 (0.2)
Gross motor skill				
≥39.26	1649 (95.5)	916 (53.0)	703 (40.7)	30 (1.7)
<39.26	78 (4.5)	42 (2.4)	32 (1.9)	4 (0.2)
Fine motor skill				
≥27.91	1620 (93.8)	899 (52.1)	690 (40.0)	31 (1.8)
<27.91	107 (6.2)	59 (3.4)	45 (2.6)	3 (0.2)
Problem‐solving skill				
≥30.03	1625 (94.1)	901 (52.2)	693 (40.1)	31 (1.8)
<30.03	102 (5.9)	57 (3.3)	42 (2.4)	3 (0.2)
Personal–social skill				
≥29.89	1685 (97.6)	933 (54.0)	720 (41.7)	32 (1.9)
<29.89	42 (2.4)	25 (1.4)	15 (0.9)	2 (0.1)

*Note*: *n* (%) or mean ± standard deviation (SD).

Abbreviations: BMI, body mass index; J‐ASQ‐3; Japanese version of the Ages and Stages Questionnaire, Third Edition.

### Association between maternal drinking and *ADH1B* and *ALDH2* gene polymorphism

Table 2A depicts the cross‐tabulation of maternal genetic polymorphisms and maternal drinking status during pregnancy. Seventy‐nine mothers (4.6%) had the ADH1B*1/*1 genotype, 616 (35.7%) had the *1/*2 genotype, and 1032 (59.8%) had the *2/*2 genotype. The *ALDH2* *1/*1, *1/*2, and *2/*2 genotypes were observed in 973 (56.3%), 653 (37.8%), and 101 (5.8%) mothers, respectively. Cross‐tabulations of alcohol consumption amount and frequency with each genotype are shown in Table 2B,C. Furthermore, Table 2D shows the cross‐tabulation results of the frequency of maternal alcohol consumption when the child was 1.5 years of age and each genotype. None of the mothers carried the ALDH2*2/*2 genotype were drinking before or during pregnancy, nor when the child was 1.5 years of age. The frequencies of *ADH1B* (*p* = 0.56) and *ALDH2* (*p* = 0.82) genotypes did not deviate significantly from the Hardy–Weinberg equilibrium.

**TABLE 2:** Association between maternal drinking status and *ADH1B* and *ALDH2* genotypes.

	Total	*ADH1B* genotype			*ALDH2* genotype		
		*1/*1	*1/*2	*2/*2	*1/*1	*1/*2	*2/*2
	1727 (100)	79 (4.6)	616 (35.7)	1032 (59.8)	973 (56.3)	653 (37.8)	101 (5.8)
(A) Maternal drinking status during pregnancy							
Never	958 (55.5)	38 (2.2)	346 (20.0)	574 (33.2)	393 (22.8)	464 (26.9)	101 (5.8)
Quit drinking in early pregnancy	735 (42.6)	39 (2.3)	259 (15.0)	437 (25.3)	551 (31.9)	184 (10.7)	0 (0.0)
Current drinker	34 (2.0)	2 (0.1)	11 (0.6)	21 (1.2)	29 (1.7)	5 (0.3)	0 (0.0)
(B) Amount of maternal alcohol consumption during pregnancy (drinks/week)							
Never	1709 (99.0)	77 (4.5)	610 (35.3)	1022 (59.2)	959 (54.5)	649 (37.6)	101 (5.8)
<1	10 (0.6)	2 (0.1)	3 (0.2)	5 (0.3)	9 (0.5)	1 (0.1)	0 (0.0)
1–7	8 (0.5)	0 (0.0)	3 (0.2)	5 (0.3)	5 (0.3)	3 (0.2)	0 (0.0)
(C) Maternal drinking frequency during pregnancy							
Never	1685 (97.6)	76 (4.4)	600 (34.7)	1009 (58.4)	938 (54.3)	646 (37.4)	101 (5.8)
Hardly	22 (1.3)	1 (0.1)	8 (0.5)	13 (0.8)	20 (1.2)	2 (0.1)	0 (0.0)
1–3 times/month	11 (0.6)	1 (0.1)	4 (0.2)	6 (0.3)	9 (0.5)	2 (0.1)	0 (0.0)
≥1–2 times/week	9 (0.5)	1 (0.1)	4 (0.2)	4 (0.2)	6 (0.3)	3 (0.2)	0 (0.0)
(D) Maternal drinking frequency when her child was 1.5 years of age							
Hardly	1210 (71.3)	54 (3.2)	437 (25.7)	719 (42.3)	577 (34.0)	533 (31.4)	100 (5.9)
1–3 times/month	185 (10.9)	8 (0.5)	65 (3.8)	112 (6.6)	135 (8.0)	50 (2.9)	0 (0.0)
1–2 times/week	134 (7.9)	1 (0.1)	45 (2.7)	88 (5.2)	105 (6.2)	29 (1.7)	0 (0.0)
≥3 times/week	169 (10.0)	15 (0.9)	53 (3.1)	101 (5.9)	143 (8.4)	26 (1.5)	0 (0.0)

*Note*: *n* (%).

### Association between maternal drinking status during pregnancy and the risk of developmental delay at 3 years of age

Table 3 displays the relationship between maternal drinking status during pregnancy and the risk of developmental delay at 3 years of age. Children of mothers who consumed alcohol during pregnancy had a higher risk of delays in communication skills when compared with those born to nondrinking mothers (cOR, 5.67; 95% CI, 1.84–17.49; aOR, 5.82; 95% CI, 1.84–18.38). Considering the other four domains of J‐ASQ‐3, children born to mothers who consumed alcohol during pregnancy had higher ORs for developmental delay than those born to nondrinking mothers, although no significant differences were detected. Children born to mothers who quit drinking in early pregnancy did not show any association with the risk of developmental delay in any of the J‐ASQ‐3 domains when compared with those born to nondrinking mothers.

**TABLE 3:** Association between maternal alcohol consumption during pregnancy and developmental delay in the offspring at 3 years of age.

Subscale of the ASQ‐3	Maternal drinking status during pregnancy	cOR (95% CI)	aOR (95% CI)
Communication	Never	Reference	Reference
	Quit drinking in early pregnancy	0.89 (0.46–1.72)	0.86 (0.44–1.68)
	Current drinkers	**5.67 (1.84–17.49)**	**5.82 (1.84–18.38)**
Gross motor	Never	Reference	Reference
	Quit drinking in early pregnancy	0.99 (0.62–1.59)	0.92 (0.57–1.49)
	Current drinkers	2.91 (0.98–8.63)	2.95 (0.97–8.93)
Fine motor	Never	Reference	Reference
	Quit drinking in early pregnancy	0.99 (0.67–1.48)	0.95 (0.64–1.43)
	Current drinkers	1.48 (0.44–4.97)	1.55 (0.45–5.31)
Problem‐solving	Never	Reference	Reference
	Quit drinking in early pregnancy	0.96 (0.64–1.45)	0.88 (0.58–1.34)
	Current drinkers	1.53 (0.45–5.16)	1.53 (0.45–5.22)
Personal–social	Never	Reference	Reference
	Quit drinking in early pregnancy	0.78 (0.41–1.49)	0.72 (0.37–1.39)
	Current drinkers	2.33 (0.53–10.28)	2.58 (0.57–11.72)

*Note*: Boldface indicates significance (*p* < 0.05). Adjusted for maternal age at birth, pre‐pregnancy body mass index, and breastfeeding until 1 year of age.

Abbreviations: aOR, adjusted odds ratio; CI, confidence interval; cOR, crude odds ratio; J‐ASQ‐3, Japanese version of the Ages and Stages Questionnaire, Third Edition.

### Association between maternal drinking status during pregnancy, maternal *ADH1B* genotype combinations, and the risk of developmental delay at 3 years of age

Table 4 illustrates whether the combination of maternal drinking status during pregnancy and maternal *ADH1B* genotype is associated with the risk of developmental delay at 3 years of age. Only two mothers carried the ADH1B*1/*1 genotype and drank alcohol during pregnancy; hence, they were grouped together as *1/*2 + *1/*1 for analysis. Considering mothers carrying the ADH1B*2/*2 genotype, children whose mothers consumed alcohol during pregnancy had an increased risk of developmental delay in communication skills when compared with those whose mothers did not drink alcohol (cOR, 7.27; 95% CI, 2.22–23.80; aOR, 7.05; 95% CI, 2.10–23.70). In the other four domains of J‐ASQ‐3, children born to mothers who drank alcohol during pregnancy had higher ORs for developmental delay than those born to nondrinking mothers, although no significant differences were detected. Considering mothers with the ADH1B*1/*1 or *1/*2 genotype, analysis was impossible because there were no cases of developmental delay in the domains of communication and personal–social skills among children born to mothers who drank alcohol during pregnancy.

**TABLE 4:** Effect of the combination of maternal drinking during pregnancy and *ADH1B* genotype on developmental delay in the offspring at 3 years of age.

Subscale of the ASQ‐3	Maternal drinking status during pregnancy	*ADH1B* genotype	Case (*n*)/Control (*n*)	cOR (95% CI)	aOR (95% CI)	*p* for interaction
Communication	Never	*2/*2	18/556	Reference	Reference	
		*1/*1 + *1/*2	4/380	**0.33 (0.11–0.97)**	0.35 (0.12–1.04)	
	Quit drinking in early pregnancy	*2/*2	8/429	0.58 (0.25–1.34)	0.57 (0.24–1.32)	
		*1/*1 + *1/*2	7/291	0.74 (0.31–1.80)	0.76 (0.31–1.86)	
	Current drinkers	*2/*2	4/17	**7.27 (2.22–23.80)**	**7.05 (2.10–23.70)**	NA
		*1/*1 + *1/*2	0/13	NA	NA	
Gross motor	Never	*2/*2	25/549	Reference	Reference	
		*1/*1 + *1/*2	17/367	1.02 (0.54–1.91)	1.04 (0.54–1.98)	
	Quit drinking in early pregnancy	*2/*2	16/421	0.84 (0.44–1.58)	0.74 (0.39–1.43)	
		*1/*1 + *1/*2	16/282	1.25 (0.66–2.37)	1.22 (0.64–2.34)	
	Current drinkers	*2/*2	3/18	**3.66 (1.01–13.25)**	3.56 (0.96–13.21)	0.64
		*1/*1 + *1/*2	1/12	1.83 (0.23–14.63)	2.03 (0.25–16.59)	
Fine motor	Never	*2/*2	30/544	Reference	Reference	
		*1/*1 + *1/*2	29/355	1.48 (0.87–2.51)	1.52 (0.89–2.61)	
	Quit drinking in early pregnancy	*2/*2	27/410	1.19 (0.70–2.04)	1.15 (0.67–1.97)	
		*1/*1 + *1/*2	18/280	1.17 (0.64–2.13)	1.13 (0.62–2.08)	
	Current drinkers	*2/*2	2/19	1.91 (0.43–8.58)	1.91 (0.42–8.72)	0.71
		*1/*1 + *1/*2	1/12	1.51 (0.19–12.01)	1.79 (0.22–14.48)	
Problem‐solving	Never	*2/*2	33/541	Reference	Reference	
		*1/*1 + *1/*2	24/360	1.09 (0.64–1.88)	1.16 (0.67–2.01)	
	Quit drinking in early pregnancy	*2/*2	23/414	0.91 (0.53–1.58)	0.84 (0.48–1.46)	
		*1/*1 + *1/*2	19/279	1.12 (0.62–2.00)	1.09 (0.61–1.96)	
	Current drinkers	*2/*2	2/19	1.73 (0.39–7.73)	1.66 (0.37–7.56)	0.87
		*1/*1 + *1/*2	1/12	1.37 (0.17–10.83)	1.55 (0.19–12.50)	
Personal–social	Never	*2/*2	20/554	Reference	Reference	
		*1/*1 + *1/*2	5/379	**0.37 (0.14–0.98)**	0.39 (0.15–1.06)	
	Quit drinking in early pregnancy	*2/*2	8/429	0.52 (0.23–1.18)	0.49 (0.21–1.13)	
		*1/*1 + *1/*2	7/291	0.67 (0.28–1.59)	0.65 (0.27–1.56)	
	Current drinkers	*2/*2	2/19	2.92 (0.64–13.38)	3.03 (0.64–14.47)	NA
		*1/*1 + *1/*2	0/13	NA	NA	

*Note*: Boldface indicates significance (*p* < 0.05). Adjusted for maternal age at birth, pre‐pregnancy body mass index, and breastfeeding until 1 year of age.

Abbreviations: aOR, adjusted odds ratio; CI, confidence interval; cOR, crude odds ratio; J‐ASQ‐3, Japanese version of the Ages and Stages Questionnaire, Third Edition; NA, not available.

### Association between maternal drinking status during pregnancy, maternal *ALDH2* genotype combinations, and the risk of developmental delay at 3 years of age

Table 5 examines the relationship between maternal drinking habits during pregnancy, maternal *ALDH2* genotype combinations, and the risk of developmental delay at 3 years of age. Mothers carrying the ALDH2*2/*2 genotype were excluded from the analysis because no participant with this genotype drank alcohol during pregnancy or postpartum. Compared with children of mothers carrying the ALDH2*1/*1 genotype who did not drink alcohol during pregnancy, children of mothers carrying the ALDH2*1/*2 genotype who drank alcohol during pregnancy had a higher risk of developmental delay in communication (aOR, 11.54; 95% CI, 1.13–118.29), gross motor (aOR, 21.70; 95% CI, 3.30–142.70), fine motor (aOR, 9.27; 95% CI, 1.44–59.88), problem‐solving (aOR, 9.41; 95% CI, 1.46–60.52), and personal–social skills (aOR, 10.63; 95% CI, 1.03–110.28). An interaction between alcohol consumption during pregnancy and *ALDH2* polymorphism on the risk of gross motor delay was observed (*p* < 0.01).

**TABLE 5:** Effect of the combination of maternal drinking during pregnancy and *ALDH2* genotype on developmental delay in the offspring at 3 years of age.

Subscale of the ASQ‐3	Maternal drinking status during pregnancy	*ALDH2* genotype	Case (*n*)/Control (*n*)	cOR (95% CI)	aOR (95% CI)	*p* for interaction
Communication	Never	*1/*1	8/385	Reference	Reference	
		*1/*2	12/452	1.28 (0.52–3.16)	1.25 (0.51–3.11)	
	Quit drinking in early pregnancy	*1/*1	9/542	0.80 (0.31–2.09)	0.79 (0.30–2.07)	
		*1/*2	6/178	1.62 (0.56–4.75)	1.55 (0.53–4.55)	
	Current drinkers	*1/*1	3/26	**5.55 (1.39–22.18)**	**5.98 (1.45–24.41)**	0.79
		*1/*2	1/4	**12.03 (1.21–120.05)**	**11.54 (1.13–118.29)**	
Gross motor	Never	*1/*1	21/372	Reference	Reference	
		*1/*2	16/448	0.63 (0.33–1.23)	0.59 (0.30–1.16)	
	Quit drinking in early pregnancy	*1/*1	21/530	0.70 (0.38–1.30)	0.60 (0.32–1.13)	
		*1/*2	11/173	1.13 (0.53–2.39)	1.01 (0.47–2.15)	
	Current drinkers	*1/*1	1/28	0.63 (0.08–4.88)	0.62 (0.08–4.82)	**< 0.01**
		*1/*2	3/2	**26.57 (4.21–167.71)**	**21.70 (3.30–142.70)**	
Fine motor	Never	*1/*1	22/371	Reference	Reference	
		*1/*2	28/436	1.08 (0.61–1.93)	1.01 (0.57–1.81)	
	Quit drinking in early pregnancy	*1/*1	29/522	0.94 (0.53–1.66)	0.85 (0.48–1.52)	
		*1/*2	16/168	1.61 (0.82–3,14)	1.45 (0.74–2.86)	
	Current drinkers	*1/*1	1/28	0.60 (0.08–4.63)	0.63 (0.08–4.93)	0.06
		*1/*2	2/3	**11.24 (1.79–70.80)**	**9.27 (1.44–59.88)**	
Problem‐solving	Never	*1/*1	22/371	Reference	Reference	
		*1/*2	28/436	1.08 (0.61–1.93)	1.03 (0.58–1.83)	
	Quit drinking in early pregnancy	*1/*1	27/524	0.87 (0.49–1.55)	0.81 (0.45–1.44)	
		*1/*2	15/169	1.50 (0.76–2.96)	1.27 (0.63–2.56)	
	Current drinkers	*1/*1	1/28	0.60 (0.08–4.63)	0.62 (0.08–4.79)	0.06
		*1/*2	2/3	**11.24 (1.79–70.80)**	**9.41 (1.46–60.52)**	
Personal–social	Never	*1/*1	8/385	Reference	Reference	
		*1/*2	15/449	1.61 (0.67–3.83)	1.51 (0.63–3.62)	
	Quit drinking in early pregnancy	*1/*1	10/541	0.89 (0.35–2.28)	0.80 (0.31–2.07)	
		*1/*2	5/179	1.34 (0.43–4.17)	1.16 (0.37–3.66)	
	Current drinkers	*1/*1	1/28	1.72 (0.21–14.23)	2.00 (0.23–17.06)	0.45
		*1/*2	1/4	**12.03 (1.21–120.05)**	**10.63 (1.03–110.28)**	

*Note*: Boldface indicates significance (*p* < 0.05). Adjusted for maternal age at birth, pre‐pregnancy body mass index, and breastfeeding until 1 year of age.

Abbreviations: aOR, adjusted odds ratio; CI, confidence interval; cOR, crude odds ratio; J‐ASQ‐3, Japanese version of the Ages and Stages Questionnaire, Third Edition; NA, not available.

## DISCUSSION

We identified an association between maternal alcohol consumption during pregnancy and an elevated risk of delayed communication skills in offspring at 3 years of age. Furthermore, in mothers carrying the ALDH2*1/*2 genotype, alcohol consumption during pregnancy was linked to an increased risk of developmental delay in five ASQ‐3 domains at 3 years of age.

In Japan, the prevalence of maternal alcohol consumption during pregnancy has exhibited a decreasing trend, dropping from 8.7% in 2010 to 4.3% in 2013 and further declining to 1.0% in 2019 (Health Japan 21, 2023). Consistent with this trend, our study recorded a rate of 2.0%. In the current study, 735 mothers (42.5%) stopped drinking after finding out that they were pregnant, while 300 mothers (28.3%) drank alcohol when their children were 1.5 years of age. These results suggest that, in Japan, alcohol consumption during pregnancy and lactation is widely understood to adversely impact the health of children.

The adverse effects on the offspring may depend on the amount of alcohol consumed during pregnancy, although the threshold remains unknown. The association between low‐dose prenatal alcohol exposure and neurodevelopmental effects or behavioral problems in offspring remains debatable. In a Danish birth cohort, low alcohol consumption (<9 drinks/week) during pregnancy did not affect attention or executive functioning in 5‐year‐old children (Skogerbø et al., 2012; Underbjerg et al., 2012). However, Lees et al. recently reported that children exposed to relatively low doses of alcohol (1–2 drinks at a time and < 7 drinks per week) throughout the prenatal period face a higher risk of developing psychological and behavioral problems during adolescence (Lees et al., 2020). All mothers in this study who drank alcohol during pregnancy were light drinkers (<7 drinks per week), and their children had greater delays in communication skills at 3 years of age. Discrepancies in these study results could be attributed to the use of diverse neurological and behavioral measures as outcome measures, as well as differences in age and confounding factors.

Maternal alcohol consumption during pregnancy is known to increase the risk of low birth weight infants (Gauthier et al., 2016). Low birth weight infants reportedly have a higher risk of neurodevelopmental abnormalities, such as cognitive impairment, motor impairment, behavioral problems, and reduced academic performance, than infants with normal birth weight (Arpi & Ferrari, 2013; Upadhyay et al., 2019; Weindrich et al., 2003). Therefore, low birth weight in infants may act as an intermediate factor in the association between maternal alcohol consumption during pregnancy and the risk of developmental delay in children. Nevertheless, in the mediation analysis, we observed that the relationship between maternal alcohol consumption during pregnancy and the increased risk of child developmental delay could directly impact communication abilities, with no indirect effect on birth weight (Table S1).

Polymorphisms in genes related to alcohol metabolism are well known to vary by ancestral populations. The frequencies of genetic polymorphisms of alcohol metabolizing enzymes have been documented in the Japanese population. Considering the *ADH1B* gene, frequencies of 5.7%, 35.4%, and 58.9% were detected for *1/*1, *1/*2, and *2/*2, respectively; for the *ALDH2* gene, the frequencies were 56.8% for *1/*1, 36.7% for *1/*2, and 6.5% for *2/*2 (Sakaue et al., 2020). Our study results revealed frequency proportions similar to those reported previously. Therefore, participants in the current study reflect a general population of females in Japan. Additionally, none of the mothers with homozygous deficiency of *ALDH2* reported drinking alcohol before or during pregnancy, suggesting the high validity of the alcohol consumption survey data collected in the current study.

The *ADH* gene, which regulates alcohol metabolism, has garnered attention owing to its role in the relationship between maternal alcohol consumption during pregnancy and the brain development of the offspring. Reportedly, mothers carrying the ADH1B*1 homozygous genotype have lower ADH activity, and prolonged fetal ethanol exposure can lead to abnormal brain development (Popova et al., 2023). Previous studies have primarily reported comparisons between mothers with the ADH1B*1 and ADH1B*3 genotypes. Several studies have reported that children of mothers with the ADH1B*1 homozygous genotype face a higher risk of FASD than those with the ADH1B*3 genotype, whereas others have documented the exact opposite result (Green & Stoler, 2007). A recent study involving children prenatally exposed to high levels of alcohol reported no association between *ADH1B* and *CYP2E1* genetic polymorphisms and the risk of FASD (Kukowka et al., 2023). Our results did not detect any effect of *ADH1B* gene polymorphism on the association between maternal alcohol consumption during pregnancy and developmental delay in offspring at 3 years of age. Ninety‐five percent of Japanese individuals carry the ADH1B*2 allele. Therefore, it is speculated that the impact of ethanol exposure on the fetus is smaller among Japanese individuals owing to the rapid metabolism of acetaldehyde from ethanol.

To the best of our knowledge, the current study is the first to demonstrate the impact of the maternal ALDH2*1/*2 genotype on the association between maternal alcohol consumption during pregnancy and the increased risk of developmental delay at 3 years of age. The ALDH2*1/*2 genotype has 17% ALDH activity when compared with the ALDH2*1/*1 genotype. Additionally, given that most Japanese people carry the ADH1B*2 allele, which results in the rapid metabolism of ethanol to acetaldehyde, a fetus of a mother carrying ALDH2*1/*2 will experience longer acetaldehyde exposure following maternal alcohol consumption during pregnancy. According to in vitro experiments, exposure to acetaldehyde induced apoptosis in rat embryos, affecting fetal development (Lee et al., 2005). Animal studies have also highlighted the importance of maternal Aldh2 in safeguarding embryos from the genotoxic effects of aldehydes (Oberbeck et al., 2014). However, the extent to which fetal acetaldehyde exposure contributes to neurodevelopmental delays in childhood remains poorly understood, necessitating further exploration of the underlying molecular mechanisms.

The strength of this study lies in the use of a Japanese birth cohort to determine the relationship between maternal drinking during pregnancy and child neurodevelopment with respect to *ADH1B* and *ALDH2* gene polymorphisms. Nevertheless, this study had some limitations. First, there is a risk of underreporting because data on alcohol consumption were collected from self‐reported questionnaires. Second, owing to the small number of mothers who consumed alcohol during pregnancy, the 95% CIs for the ORs were wide, and the precision of the estimates was not high. Additionally, the effects of the amount and frequency of alcohol consumption could not be analyzed. Therefore, further investigation with larger sample sizes is necessary in the future. Third, in this study, the logistic regression analysis was conducted with reference to previous studies, and no corrections for multiple testing were applied to the five subdomains of the ASQ‐3 (Avalos et al., 2020; Hirata et al., 2022; Motoki et al., 2022). Therefore, the possibility of false positives in these results needs to be considered. Fourth, although our analyses were adjusted for confounding factors identified by previous studies, such as maternal age, maternal BMI, and breastfeeding when the child was 1 year old, the possibility of residual confounding cannot be eliminated. Given the small number of cases in the outcomes, we were unable to adjust for numerous confounding factors. However, sensitivity analysis adding one additional confounder (maternal smoking, household income, sex of the child, and presence of siblings) yielded similar results. Fifth, in this study, the number of mothers who consumed alcohol during pregnancy carrying the ADH1B*1/ALDH2*2 polymorphism combination was limited (Table S2), thus preventing the analysis of the combined effects of *ADH1B* and *ALDH2* genotypes. Sixth, the maternal blood samples used for genome analysis were collected when the children were 8 years of age, which may have introduced selection bias as they were volunteers who cooperated in an additional survey. Moreover, the influence of alcohol metabolism‐related gene polymorphisms in children could not be evaluated.

## CONCLUSIONS

Even in the presence of a genotype with high alcohol metabolism activity, maternal drinking during pregnancy was linked to an increased risk of communication delay in the offspring. Additionally, we found that maternal alcohol consumption during pregnancy among those with the ALDH2*1/*2 genotype increases the risk of developmental delay in all domains of the J‐ASQ‐3 at 3 years of age. Regardless of the alcohol metabolism genotype, alcohol intake during pregnancy should be avoided entirely to ensure the healthy development of the child. Additional longitudinal studies are necessary to elucidate the persistent effects of maternal alcohol consumption on offspring development.

## FUNDING INFORMATION

This research received support from a Grant‐in‐Aid for Scientific Research conferred by the Japanese Ministry of Education, Culture, Sports, Science, and Technology (no.: JP20H03928) and the Osake‐no‐Kagaku Foundation. The JECS was funded by the Ministry of the Environment, Japan. The findings and conclusions of this study are solely the responsibility of the authors and do not represent the official views of the above‐mentioned governments.

## CONFLICT OF INTEREST STATEMENT

The authors declare no conflict of interest.

## Supporting information


Appendix S1.

## Data Availability

Data are unsuitable for public deposition due to ethical restrictions and the legal framework of Japan. It is prohibited by the Act on the Protection of Personal Information to publicly deposit the data containing personal information. Ethical Guidelines for Medical and Health Research Involving Human Subjects, enforced by the Japan Ministry of Education, Culture, Sports, Science and Technology and the Ministry of Health, Labor and Welfare, also restrict the open sharing of the epidemiologic data. All inquiries regarding access to data should be addressed to Dr. Shoji Nakayama, JECS Program Office Manager at National Institute for Environmental Studies (jecs-en@nies.go.jp).

## References

[acer15487-bib-0001] Arpi, E. & Ferrari, F. (2013) Preterm birth and behaviour problems in infants and preschool‐age children: a review of the recent literature. Developmental Medicine and Child Neurology, 55, 788–796. 23521214 10.1111/dmcn.12142

[acer15487-bib-0002] Avalos, L.A. , Ferber, J. , Zerbo, O. , Naleway, A.L. , Bulkley, J. , Thompson, M. et al. (2020) Trivalent inactivated influenza vaccine (IIV3) during pregnancy and six‐month infant development. Vaccine, 38, 2326–2332. 32033850 10.1016/j.vaccine.2020.01.059 PMC7309563

[acer15487-bib-0003] Black, M.M. , Walker, S.P. , Fernald, L.C.H. , Andersen, C.T. , DiGirolamo, A.M. , Lu, C. et al. (2017) Early childhood development coming of age: science through the life course. Lancet, 389, 77–90. 27717614 10.1016/S0140-6736(16)31389-7 PMC5884058

[acer15487-bib-0004] Brennan, P. , Lewis, S. , Hashibe, M. , Bell, D.A. , Boffetta, P. , Bouchardy, C. et al. (2004) Pooled analysis of alcohol dehydrogenase genotypes and head and neck cancer: a HuGE review. American Journal of Epidemiology, 159, 1–16. 14693654 10.1093/aje/kwh003

[acer15487-bib-0005] Chambers, C.D. , Coles, C. , Kable, J. , Akshoomoff, N. , Xu, R. , Zellner, J.A. et al. (2019) Fetal alcohol spectrum disorders in a Pacific Southwest City: maternal and child characteristics. Alcoholism, Clinical and Experimental Research, 43, 2578–2590. 31688971 10.1111/acer.14213 PMC6904497

[acer15487-bib-0006] Denny, L. , Coles, S. & Blitz, R. (2017) Fetal alcohol syndrome and fetal alcohol spectrum disorders. American Family Physician, 96, 515–522. 29094891

[acer15487-bib-0007] Du Plooy, C.P. , Malcolm‐Smith, S. , Adnams, C.M. , Stein, D.J. & Donald, K.A. (2016) The effects of prenatal alcohol exposure on episodic memory functioning: a systematic review. Archives of Clinical Neuropsychology, 31, 710–726. 27600446 10.1093/arclin/acw067

[acer15487-bib-0008] Edenberg, H.J. & McClintick, J.N. (2018) Alcohol dehydrogenases, aldehyde dehydrogenases, and alcohol use disorders: a critical review. Alcoholism, Clinical and Experimental Research, 42, 2281–2297. 30320893 10.1111/acer.13904 PMC6286250

[acer15487-bib-0009] Eng, M.Y. , Luczak, S.E. & Wall, T.L. (2007) ALDH2, ADH1B, and ADH1C genotypes in Asians: a literature review. Alcohol Research & Health, 30, 22–27. 17718397 PMC3860439

[acer15487-bib-0010] Gauthier, T.W. , Guidot, D.M. , Kelleman, M.S. , McCracken, C.E. & Brown, L.A. (2016) Maternal alcohol use during pregnancy and associated morbidities in very low birth weight newborns. American Journal of the Medical Sciences, 352, 368–375. 27776718 10.1016/j.amjms.2016.06.019 PMC5098418

[acer15487-bib-0011] Green, R.F. & Stoler, J.M. (2007) Alcohol dehydrogenase 1B genotype and fetal alcohol syndrome: a HuGE minireview. American Journal of Obstetrics and Gynecology, 197, 12–25. 17618743 10.1016/j.ajog.2007.02.028

[acer15487-bib-0012] Health Japan 21 (Japan) . (2023) Ministry of Health, Labour and Welfare of Japan . Available at: https://www.nibiohn.go.jp/eiken/kenkounippon21/en/kenkounippon21/genjouchi.html#Table05 [Accessed 6th November 2023].

[acer15487-bib-0013] Hirata, K. , Ueda, K. , Wada, K. , Ikehara, S. , Tanigawa, K. , Kimura, T. et al. (2022) Long‐term outcomes of children with neonatal transfer: the Japan environment and Children's study. European Journal of Pediatrics, 181, 2501–2511. 35333975 10.1007/s00431-022-04450-7 PMC9889501

[acer15487-bib-0014] Howe, L.J. , Sharp, G.C. , Hemani, G. , Zuccolo, L. , Richmond, S. & Lewis, S.J. (2019) Prenatal alcohol exposure and facial morphology in a UK cohort. Drug and Alcohol Dependence, 197, 42–47. 30772781 10.1016/j.drugalcdep.2018.11.031

[acer15487-bib-0015] Jones, K.L. & Smith, D.W. (1973) Recognition of the fetal alcohol syndrome in early infancy. Lancet, 302, 999–1001. 4127281 10.1016/s0140-6736(73)91092-1

[acer15487-bib-0016] Kawamoto, T. , Nitta, H. , Murata, K. , Toda, E. , Tsukamoto, N. , Hasegawa, M. et al. (2014) Rationale and study design of the Japan environment and children's study (JECS). BMC Public Health, 14, 25. 24410977 10.1186/1471-2458-14-25 PMC3893509

[acer15487-bib-0017] Kesmodel, U.S. , Nygaard, S.S. , Mortensen, E.L. , Bertrand, J. , Denny, C.H. , Glidewell, A. et al. (2019) Are low‐to‐moderate average alcohol consumption and isolated episodes of binge drinking in early pregnancy associated with facial features related to fetal alcohol syndrome in 5‐year‐old children? Alcoholism, Clinical and Experimental Research, 43, 1199–1212. 30977899 10.1111/acer.14047 PMC6691727

[acer15487-bib-0018] Kitagawa, K. , Kawamoto, T. , Kunugita, N. , Tsukiyama, T. , Okamoto, K. , Yoshida, A. et al. (2000) Aldehyde dehydrogenase (ALDH) 2 associates with oxidation of methoxyacetaldehyde; in vitro analysis with liver subcellular fraction derived from human and Aldh2 gene targeting mouse. FEBS Letters, 476, 306–311. 10913633 10.1016/s0014-5793(00)01710-5

[acer15487-bib-0019] Kukowka, A. , Brzuchalski, B. , Kurzawski, M. , Malinowski, D. & Białecka, M.A. (2023) ADH1B, ADH1B/C and CYP2E1 gene polymorphism and the risk of fetal alcohol spectrum disorder. Genes, 14, 1392. 37510297 10.3390/genes14071392 PMC10379323

[acer15487-bib-0020] Kurita, H. , Motoki, N. , Inaba, Y. , Misawa, Y. , Ohira, S. , Kanai, M. et al. (2021) Maternal alcohol consumption and risk of offspring with congenital malformation: the Japan environment and Children's study. Pediatric Research, 90, 479–486. 33230193 10.1038/s41390-020-01274-9 PMC8460444

[acer15487-bib-0021] Lee, R.D. , An, S.M. , Kim, S.S. , Rhee, G.S. , Kwack, S.J. , Seok, J.H. et al. (2005) Neurotoxic effects of alcohol and acetaldehyde during embryonic development. Journal of Toxicology and Environmental Health, Part A, 68, 2147–2162. 16326430 10.1080/15287390500177255

[acer15487-bib-0022] Lees, B. , Mewton, L. , Jacobus, J. , Valadez, E.A. , Stapinski, L.A. , Teesson, M. et al. (2020) Association of prenatal alcohol exposure with psychological, behavioral, and neurodevelopmental outcomes in children from the adolescent brain cognitive development study. American Journal of Psychiatry, 177, 1060–1072. 32972200 10.1176/appi.ajp.2020.20010086 PMC7924902

[acer15487-bib-0023] Little, R.E. , Northstone, K. , Golding, J. & ALSPAC Study Team . (2002) Alcohol, breastfeeding, and development at 18 months. Pediatrics, 109, e72. 11986478 10.1542/peds.109.5.e72

[acer15487-bib-0024] Maher, G.M. , Khashan, A.S. , O'Byrne, L. , Flanagan, S. , Mortimer, R.M. , Kiely, M. et al. (2022) Periconceptual and prenatal alcohol consumption and neurodevelopment at age two and five years. European Journal of Obstetrics & Gynecology and Reproductive Biology, 274, 197–203. 35667175 10.1016/j.ejogrb.2022.05.034

[acer15487-bib-0025] Mezawa, H. , Aoki, S. , Nakayama, S.F. , Nitta, H. , Ikeda, N. , Kato, K. et al. (2019) Psychometric profile of the ages and stages questionnaires, Japanese translation. Pediatrics International, 61, 1086–1095. 31419360 10.1111/ped.13990 PMC6899956

[acer15487-bib-0026] Michikawa, T. , Nitta, H. , Nakayama, S.F. , Yamazaki, S. , Isobe, T. , Tamura, K. et al. (2018) Baseline profile of participants in the Japan environment and Children's study (JECS). Journal of Epidemiology, 28, 99–104. 29093304 10.2188/jea.JE20170018 PMC5792233

[acer15487-bib-0027] Miyake, K. , Mochizuki, K. , Kushima, M. , Shinohara, R. , Horiuchi, S. , Otawa, S. et al. (2023) Maternal protein intake in early pregnancy and child development at age 3 years. Pediatric Research, 94, 392–399. 36624288 10.1038/s41390-022-02435-8

[acer15487-bib-0028] Motoki, N. , Inaba, Y. , Shibazaki, T. , Misawa, Y. , Ohira, S. , Kanai, M. et al. (2022) Insufficient maternal gestational weight gain and infant neurodevelopment at 12 months of age: the Japan environment and Children's study. European Journal of Pediatrics, 181, 921–931. 34642790 10.1007/s00431-021-04232-7 PMC8897327

[acer15487-bib-0029] Oberbeck, N. , Langevin, F. , King, G. , de Wind, N. , Crossan, G.P. & Patel, K.J. (2014) Maternal aldehyde elimination during pregnancy preserves the fetal genome. Molecular Cell, 55, 807–817. 25155611 10.1016/j.molcel.2014.07.010 PMC4175174

[acer15487-bib-0030] O'Callaghan, F.V. , O'Callaghan, M. , Najman, J.M. , Williams, G.M. & Bor, W. (2007) Prenatal alcohol exposure and attention, learning and intellectual ability at 14 years: a prospective longitudinal study. Early Human Development, 83, 115–123. 16842939 10.1016/j.earlhumdev.2006.05.011

[acer15487-bib-0031] Panczakiewicz, A.L. , Glass, L. , Coles, C.D. , Kable, J.A. , Sowell, E.R. , Wozniak, J.R. et al. (2016) Neurobehavioral deficits consistent across age and sex in youth with prenatal alcohol exposure. Alcoholism, Clinical and Experimental Research, 40, 1971–1981. 27430360 10.1111/acer.13153 PMC5008991

[acer15487-bib-0032] Polimanti, R. & Gelernter, J. (2018) ADH1B: from alcoholism, natural selection, and cancer to the human phenome. American Journal of Medical Genetics Part B: Neuropsychiatric Genetics, 177, 113–125. 10.1002/ajmg.b.32523 PMC5617762 28349588

[acer15487-bib-0033] Popova, S. , Charness, M.E. , Burd, L. , Crawford, A. , Hoyme, H.E. , Mukherjee, R.A.S. et al. (2023) Fetal alcohol spectrum disorders. Nature Reviews Disease Primers, 9, 11. 10.1038/s41572-023-00420-x 36823161

[acer15487-bib-0034] Popova, S. , Lange, S. , Probst, C. , Gmel, G. & Rehm, J. (2017) Estimation of national, regional, and global prevalence of alcohol use during pregnancy and fetal alcohol syndrome: a systematic review and meta‐analysis. The Lancet Global Health, 5, e290–e299. 28089487 10.1016/S2214-109X(17)30021-9

[acer15487-bib-0035] Sakaue, S. , Akiyama, M. , Hirata, M. , Matsuda, K. , Murakami, Y. , Kubo, M. et al. (2020) Functional variants in ADH1B and ALDH2 are non‐additively associated with all‐cause mortality in Japanese population. European Journal of Human Genetics, 28, 378–382. 31558841 10.1038/s41431-019-0518-y PMC7028931

[acer15487-bib-0036] Skogerbø, Å. , Kesmodel, U.S. , Wimberley, T. , Støvring, H. , Bertrand, J. , Landrø, N.I. et al. (2012) The effects of low to moderate alcohol consumption and binge drinking in early pregnancy on executive function in 5‐year‐old children. BJOG, 119, 1201–1210. 22712874 10.1111/j.1471-0528.2012.03397.x PMC4469190

[acer15487-bib-0037] Underbjerg, M. , Kesmodel, U.S. , Landrø, N.I. , Bakketeig, L. , Grove, J. , Wimberley, T. et al. (2012) The effects of low to moderate alcohol consumption and binge drinking in early pregnancy on selective and sustained attention in 5‐year‐old children. BJOG: An International Journal of Obstetrics & Gynaecology, 119, 1211–1221. 22712829 10.1111/j.1471-0528.2012.03396.x

[acer15487-bib-0038] Upadhyay, R.P. , Naik, G. , Choudhary, T.S. , Chowdhury, R. , Taneja, S. , Bhandari, N. et al. (2019) Cognitive and motor outcomes in children born low birth weight: a systematic review and meta‐analysis of studies from South Asia. BMC Pediatrics, 19, 35. 30696415 10.1186/s12887-019-1408-8 PMC6350290

[acer15487-bib-0039] Wall, T.L. , Luczak, S.E. & Hiller‐Sturmhöfel, S. (2016) Biology, genetics, and environment: underlying factors influencing alcohol metabolism. Alcohol Research: Current Reviews, 38, 59–68. 27163368 10.35946/arcr.v38.1.08 PMC4872614

[acer15487-bib-0040] Weindrich, D. , Jennen‐Steinmetz, C. , Laucht, M. & Schmidt, M.H. (2003) Late sequelae of low birthweight: mediators of poor school performance at 11 years. Developmental Medicine and Child Neurology, 45, 463–469. 12828400 10.1017/s0012162203000860

[acer15487-bib-0041] Yokoyama, Y. , Takachi, R. , Ishihara, J. , Ishii, Y. , Sasazuki, S. , Sawada, N. et al. (2016) Validity of short and long self‐administered food frequency questionnaires in ranking dietary intake in middle‐aged and elderly Japanese in the Japan public health center‐based prospective study for the next generation (JPHC‐NEXT) protocol area. Journal of Epidemiology, 26, 420–432. 27064130 10.2188/jea.JE20150064 PMC4967663

