# Supplementary material for: Maternal alcohol consumption during pregnancy and child development: Role of ADH1B and ALDH2 gene polymorphisms—The Yamanashi Adjunct Study of the Japan Environment and Children's Study
Source: Alcohol Clin Exp Res (Hoboken). 2024 Nov 13;49(1):117–27. doi: 10.1111/acer.15487 (PMC11740163; doi:10.1111/acer.15487)
Supplement: Supplementary file 1 — Appendix S1. [file ACER-49-117-s001.docx]

**Supplementary information**

A mediation analysis was used to estimate the degree of the association between maternal alcohol consumption during pregnancy and neurodevelopmental delay at age 3 years, which is explained by birth weight. The direct effect was the effect of the exposure (X) on the outcome (Y) at a fixed level of the mediator (M). The indirect effect of X on Y through M can be quantified as the product of two coefficients: a (the effect of X on M) and b (the effect of M on Y) pathways (i.e., ab).

Herein, the exposure was the maternal alcohol consumption status during pregnancy (never drank, quit drinking in early pregnancy, current drinkers); the outcome was the presence or absence of developmental delay in each domain of the ASQ-3 assessment for 3-year-old children; and the mediator was the birth weight (g).

Mediation analysis included maternal age at birth, pre-pregnancy BMI, breastfeeding until 1 year of age as covariates. The bias-corrected and accelerated CIs of the indirect effect (ab) were calculated by bootstrapping with 5,000 iterations. Mediation analysis was performed by using PROCESS version 3.5, a macro implemented in SPSS (IBM, Armonk, NY, USA). We present the comparison between children born to mothers who did not drink alcohol during pregnancy and those born to mothers who continued drinking during pregnancy in Table S1.

**TABLE S1.** A mediation analysis of the effect of birth weight on the association between maternal alcohol consumption during pregnancy and neurodevelopmental delay at 3 years of age

| Subscales in ASQ-3 |  | Direct effect (c' path) | | Indirect effect | |
| --- | --- | --- | --- | --- | --- |
|  |  | β | 95 % CI | β | 95 % CI |
| Communication | Crude | **1.77** | **(0.58, 3.05)** | -0.004 | (-0.20, 0.18) |
|  | Adjusted | **1.83** | **(0.67, 2.99)** | 0.01 | (-0.17, 0.18) |
| Gross Motor | Crude | 1.09 | (-0.06, 2.19) | -0.004 | (-0.17, 0.15) |
|  | Adjusted | 1.08 | (-0.05, 2.20) | 0.01 | (-0.15, 0.17) |
| Fine Motor | Crude | 0.40 | (-0.82, 1.62) | -0.003 | (-0.15, 0.14) |
|  | Adjusted | 0.42 | (-0.82, 1.65) | 0.009 | (-0.13, 0.14) |
| Problem Solving | Crude | 0.43 | (-0.79, 1.65) | -0.003 | (-0.14, 0.12) |
|  | Adjusted | 0.41 | (-0.82, 1.64) | 0.008 | (-0.12, 0.13) |
| Personal-Social | Crude | 0.89 | (-0.61, 2.38) | -0.005 | (-0.22, 0.19) |
|  | Adjusted | 0.95 | (-0.58, 2.47) | 0.01 | (-0.19, 0.20) |

CI, confidence interval; ASQ-3, Ages and Stages Questionnaires, Third Edition. Boldface indicates significance (p <0.05). β represents the regression coefficient.

Adjusted for maternal age at birth, pre-pregnancy BMI, breastfeeding until 1 year of age.

**TABLE S2.** The distribution of maternal alcohol consumption during pregnancy based on combinations of *ALDH2* and *ADH1B* polymorphisms.

| **Genotype** | | **Maternal drinking status during pregnancy** | | |  |
| --- | --- | --- | --- | --- | --- |
| *ADH1B* | *ALDH2* | Never | Quit drinking in early pregnancy | Current drinker | Total |
| *2/*2 | *1/*1 | 243 (14.1) | 337 (19.5) | 18 (1.0) | 598 (34.6) |
| *2/*2 | *1/*2 | 280 (16.2) | 100 (5.8) | 3 (0.2) | 383 (22.2) |
| *2/*2 | *2/*2 | 51 (3.0) | 0 (0) | 0 (0) | 51 (3.0) |
| *1/*2 | *1/*1 | 131 (7.6) | 188 (4.1) | 9 (0.5) | 328 (19.0) |
| *1/*2 | *1/*2 | 170 (9.8) | 71 (4.1) | 2 (0.1) | 243 (14.1) |
| *1/*2 | *2/*2 | 45 (2.6) | 0 (0) | 0 (0) | 45 (2.6) |
| *1/*1 | *1/*1 | 19 (1.1) | 26 (1.5) | 2 (0.1) | 47 (2.7) |
| *1/*1 | *1/*2 | 14 (0.8) | 13 (0.8) | 0 (0) | 27 (1.6) |
| *1/*1 | *2/*2 | 5 (0.3) | 0 (0) | 0 (0) | 5 (0.3) |
| Total | | 958 (55.5) | 735 (42.6) | 34 (2.0) | 1,727 (100) |

n (%)
